# Comment on “Impact of the Surgical Approach for Neoadjuvantly Treated Gastroesophageal Junction Type II Tumors: A Multinational, High-Volume Center Retrospective Cohort Analysis”

**DOI:** 10.1097/AS9.0000000000000446

**Published:** 2024-05-22

**Authors:** Arnar B. Ingason, Mitchell C. Norotsky

**Affiliations:** * From the Department of Surgery, University of Vermont Larner College of Medicine, Burlington, VT.

We read the recent publication by Wirsik et al with interest. It showed that patients undergoing transthoracic esophagectomies for Siewert type II gastroesophageal junction (GEJ) cancer had higher overall survival and R0 resection rates compared with patients who underwent transhiatal gastrectomies.^1^ The authors concluded that while awaiting the results of randomized controlled trials, their study may guide treatment selection for patients with type II GEJ tumors.

In our opinion, the results of the study by Wirsik et al must be interpreted in the context of a few important limitations. First, the study does not adequately account for patient comorbidities. Obviously, there are many factors that go into the decision on whether to proceed with a transhiatal or transthoracic approach. Patients with advanced lung disease may, for example, not be ideal candidates for transthoracic surgery. Other underlying diseases, such as diabetes, coronary artery disease, congestive heart failure, and chronic kidney disease, may affect a patient’s risk of postoperative complications and the prevalence of these factors may be different in the 2 surgical groups. While the authors did include the American Society of Anesthesiologists score in their propensity score model (which in itself is limited as >95% of patients in the study had a score of 2 or 3), the study does not account for overall comorbidity burden or individual diseases, and this increases the risk of selection bias. Patient frailty is another factor that is strongly associated with postoperative outcomes^2^ and was not accounted for in the study. While a patient’s frailty is usually obvious at the bedside, it can be difficult to appreciate on paper. However, validated frailty risk scores, such as the Hospital Frailty Risk Score, have been created.^3^ Additionally, the study would have benefitted from accounting for concomitant drug use. For example, preoperative use of opioids and benzodiazepines has been associated with higher postoperative mortality.^4^

Second, while the exposure of interest is transhiatal versus transthoracic surgery, there was significant variation in the way patients were treated before surgery, and this was not adequately accounted for. For example, 9% of patients undergoing transhiatal surgery received chemoradiation and 87% received chemotherapy only. Meanwhile, 46% of patients undergoing transthoracic surgery received chemoradiation. Unfortunately, this was not accounted for in the propensity score model. The authors did perform a sensitivity analysis including only patients that received chemotherapy without radiation. This analysis also demonstrated superior outcomes for patients undergoing transthoracic approach. However, as far as we can tell, this analysis was only performed using unadjusted data and not propensity score modeling. Given this, we are concerned that the difference in outcomes between patients undergoing transhiatal and transthoracic approaches may, at least partly, be explained by differences in neoadjuvant treatment. Supporting this, the operative pathology demonstrated that only 2.7% of patients undergoing transthoracic approach had distant metastasis at the time of surgery, compared with 11.4% of patients undergoing transhiatal approach. Similarly, complete pathological response was noted in 14.8% of patients undergoing transthoracic surgery compared with 9.1% of patients undergoing transhiatal surgery. These differences are unrelated to the surgery itself and instead suggest that patients undergoing transthoracic surgery had either (1) better response to their neoadjuvant therapy or (2) less advanced disease at baseline compared with their counterparts undergoing transhiatal surgery.

Third, patients included in the study underwent either open, minimally invasive, hybrid, or robotic-assisted surgical approaches. Surgical outcomes may vary for patients undergoing these different approaches and this should have been accounted for in the study. Supporting this, a meta-analysis including 130 studies found that open transthoracic surgery was associated with higher postoperative mortality compared with both minimally invasive and hybrid transthoracic surgery.^5^

Finally, the study included 6 different centers with an unreported number of different surgeons performing the procedures. As there may be a predilection for one surgical approach over the other at individual centers, and as surgical outcomes may differ significantly between individual centers, this should have been accounted for in the propensity score model.

In summary, it is, in our opinion, unclear whether the improved survival found for patients undergoing transthoracic esophagectomies compared to transhiatal gastrectomies, in the study by Wirsik et al, is related to the surgery itself or by differences in patient selection and neoadjuvant chemotherapy provided to the 2 groups. Until the results of the CARDIA trial are published, designed to compare transthoracic and transhiatal surgeries for type II GEJ cancer,^6^ there is inadequate evidence to support the use of one approach over the other.
